# Immune-Mediated Hepatitis During Immune Checkpoint Inhibitor cancer Immunotherapy: Lessons From Autoimmune Hepatitis and Liver Immunology

**DOI:** 10.3389/fimmu.2022.907591

**Published:** 2022-06-30

**Authors:** Julian Hercun, Catherine Vincent, Marc Bilodeau, Pascal Lapierre

**Affiliations:** ^1^ Département de médecine, Université de Montréal, Montréal, QC, Canada; ^2^ Centre de recherche du Centre Hospitalier de l’Université de Montréal (CRCHUM), Université de Montréal, Montréal, QC, Canada

**Keywords:** immunotherapy, cancer, hepatitis, immune-related adverse event, liver

## Abstract

Immune checkpoint inhibitors (ICI) are being increasingly used to successfully treat several types of cancer. However, due to their mode of action, these treatments are associated with several immune-related adverse events (irAEs), including immune-mediated autoimmune-like hepatitis in 5 to 10% of cases. The specific immune mechanism responsible for the development of immune-mediated liver injury caused by immune checkpoint inhibitors (ILICI) is currently unknown. This review summarizes the current knowledge on hepatic irAEs during cancer immunotherapy. It also addresses the clinical management of ILICI and how it is becoming an increasingly important clinical issue. Clinical, histological, and laboratory features of autoimmune hepatitis (AIH) and ILICI are compared, and their shared and distinctive traits are discussed in an effort to better understand the development of hepatic irAEs. Finally, based on the current knowledge of liver immunology and AIH pathogenesis, we propose a series of events that could trigger the observed liver injury in ICI-treated patients. This model could be useful in the design of future studies aiming to identify the specific immune mechanism(s) at play in ILICI and improve immune checkpoint inhibitor cancer immunotherapy.

## Introduction

Very little is known about the origins of immune checkpoint inhibitor (ICI) hepatitis. Liver toxicity depends on the type of immunotherapy administered, the dose, and the existence of pre-existing liver conditions. The incidence of ICI hepatitis, now referred to as immune-mediated liver injury caused by immune checkpoint inhibitors (ILICI) (1), is higher in patients who receive combination therapy than in those on monotherapy (2–4). A pre-existing liver condition such as cirrhosis also increases the risk of hepatotoxicity in patients treated for hepatocellular carcinoma (HCC) (5, 6). However, the underlying responsible immune mechanism(s) are unknown.

Several mechanisms have been proposed to explain irAEs and ILICI. First, direct cytotoxicity of the administered antibodies through complement activation has been suggested (7). This is supported by the fact that PD-1 and PD-L1, the target of several cancer immunotherapies, are also expressed by healthy tissue (7). An early change in circulating B cells has also been correlated with the frequency and timing of irAEs (8). However, these proposed mechanisms link immunotherapies with irAEs but none of these are specific to the liver and could explain how cancer immunotherapies could lead to ILICI.

Herein, we will review the current knowledge on cancer immunotherapy and hepatic irAEs and factors that can influence the incidence of ILICI. We will then propose a sequence of events that could lead to ILICI based on current knowledge of liver immunology and the pathogenesis of autoimmune hepatitis (AIH). This putative model could serve as the basis for future studies aiming at reducing the occurrence of hepatic irAEs and improving our knowledge of the pathogenesis of ILICI.

## Cancer Immunotherapy

The use of ICI was first approved by the United States Federal Drug Administration and European Medicines Agency in 2011 following the results of anti-CTLA-4 therapy in metastatic melanoma (9). Since this milestone moment in cancer therapeutics, ICIs have been granted approval by regulatory agencies for multiple other cancers (**Table 1**) (5). There are currently over 120 phase 3 studies actively recruiting on the National Library of Medicine’s Clinical Trials website for previously approved indications but also for other gastro-intestinal cancers, leukemia, endometrial cancer, brain cancer, and ovarian cancer.

**Table 1:** Immune checkpoint inhibitors approved by US and European regulatory authorities.

AGENT	TARGET		INDICATION	FDA approval	EMA approval
atezolizumab (Tecentriq)		PD-L1	melanoma, small cell and non-small cell lung cancer, hepatocellular carcinoma. urothelial carcinoma	2016	2017
ipilimumab (Yervoy)		CTLA-4	Melanoma, renal cell carcinoma, MSI high or MMR deficient colorectal cancer, hepatocellular carcinoma, non-small cell lung cancer, mesothelioma	2011	2011
nivolumab (Opdivo)		PD-1	melanoma, renal cell cancer, non-small cell lung cancer, Hepatocellular carcinoma, Head and neck squamous cell carcinoma, urothelial carcinoma, esophageal or gastric cancer, colorectal cancer, Hodgkin lymphoma, mesothelioma	2014	2015
pembrolizumab (Keytruda)		PD-L1	melanoma, non-small cell lung cancer, head and neck squamous cell carcinoma, urothelial carcinoma, gastric adenocarcinoma, MSI high or MMR deficient colorectal cancer, Hodgkin lymphoma, cervical cancer, renal carcinoma, Merkel cell carcinoma, cutaneous squamous cell carcinoma, triple-negative breast cancer	2014	2015
Avelumab (Bavencio)		PD-L1	Merkel Cell Carcinoma, urothelial carcinoma, renal cell carcinoma	2017	2017
Durvalumab (Imfinzi)		PD-L1	Small cell and non-small cell lung cancer	2017	2018
Cemiplimab (Libtayo)		PD-1	Non-small cell lung cancer, cutaneous squamous cell carcinoma, Basal cell carcinoma	2018	2019

FDA, United States Federal Drug Administration; EMA, European Medicines Agency; PD-1, Programmed cell death protein 1; PD-L1, Programmed death-ligand 1, CTLA-4; Cytotoxic T-lymphocyte associated protein 4, MSI; Microsatellite instability, MMR; mismatch repair.

### Immune-Related Adverse Events

During initial trials, a specific immune-mediated pattern of toxicity associated with ICI was reported and termed *immune-related adverse events* (irAEs). The skin, colon, liver, lungs, and endocrine organs are the most commonly affected sites; however rarer cases of cardiac or neurological irAEs are associated with severe adverse outcomes (10). Over time, an increased incidence has been reported, likely due to the increased use of ICI (0.9 cases/month to 19.25 cases/month between 2015 and 2018) (11). Toxicity is graded on the *Common Terminology Criteria for Adverse Events* (CTCAE) scale with grade 5 representing death. Reporting in oncology clinical trials is based on this scale, where severe events are defined as ≥ grade 3. Overall, severe irAEs involve 10-27% of patients in studies (12). The grading of hepatic adverse events is described in **Table 2**.

**Table 2:** CTCAE Grading of hepatic adverse events.

	Grade 1	Grade 2	Grade 3	Grade 4	Grade 5
Alanine aminotransferase (ALT)	>ULN - 3x ULN	3-5 x ULN	5-20 x ULN	>20 x ULN	Death
Aspartate aminotransferase (AST)	>ULN - 3x ULN	3-5 x ULN	5-20 x ULN	>20 x ULN	Death
Total Bilirubin	>ULN - 1.5 x ULN	1.5-3 x ULN	3-10 x ULN	>10 x ULN	Death
Alkaline phosphatase (ALP)	>ULN – 2.5 x ULN	2.5-5 x ULN	5-20 x ULN	>20 x ULN	Death

### Hepatic Immune-Related Adverse Events

Hepatic irAEs are a clinically relevant entity with an incidence of 5-10% in single-agent ICI therapy; however, severe toxicity occurs in less than 2% of cases but with increased incidence in combination therapy (12). Cases of ILICI present as an acute rise followed by a progressive and rapid decrease of liver enzymes, predominantly transaminases (ALT and AST) (13). Onset is most often between 6 to 12 weeks after treatment initiation (14). Elevation of liver enzymes on ICI therapy is common and other etiologies should not be overlooked; while liver injury occurred in 14% of patients in one cohort, only 28% of cases could be attributed to ILICI, with greater toxicity in patients with pre-treatment liver metastases (15). In another retrospective study, ILICI accounted only for 46% of cases of elevated liver enzymes beyond grade 2 with multiple other causes identified (16).

Besides excluding other causes, assessment of ILICI potentially requires hepatic imaging and histological assessment. Imaging findings are non-specific and include hepatomegaly, peri-portal edema, and lymphadenopathy, with more prominent findings in cases of severe hepatitis (17). Histological assessment reveals acute hepatitis, as is seen in cases of viral hepatitis, AIH, or drug-induced liver injury (18). The pattern is most often described as panlobular or restricted to zone 3 with lesser involvement of the periportal area. A mixed cellular infiltrate with lymphocytes (mostly CD8+ T cells), plasma cells, or eosinophils is seen (19). A specific histological pattern has been described for CTLA-4-induced toxicity characterized by granulomatous hepatitis with fibrin ring granulomas and central vein endotheliitis (20).

ILICI shares several histological traits with AIH such as hepatitis with a panlobular distribution, presence of hepatocellular necrosis, and a lymphocytic infiltrate. These findings are distinct from classic AIH through the presence of histiocytic sinusoidal infiltrates, microgranulomas, and central vein endotheliitis and by the absence of a consistent plasma cell predominant infiltrate (17, 21). Additionally, the increased presence of CD8+ T cells with a lesser proportion of CD4+ T cells in ILICI can assist in differentiating it from AIH (19, 22).

While the predominant pattern of histological liver injury is hepatitis, a biliary pattern has also been observed, albeit less commonly, and is usually associated with a higher metastatic liver burden or other causes of liver injury (21). This pattern has been mostly reported with PD-1 antagonist toxicity, typically presenting as acute cholangitis or vanishing bile duct syndrome and with a poor response to immunosuppressive therapy (23, 24).

### Incidence of Hepatic irAEs

The initial reports on the occurrence of ILICI were reassuring: in the initial phase 2 study of ipilimumab for advanced melanoma, liver adverse events ≥ grade 3 occurred in 3%, with complete resolution of all liver-related AEs (25). The FDA licensing study reported AST elevations in 0.8% and ALT elevations in 1.5% of patients without any cases beyond grade 3 (9). This contrasts with reports of a greater burden of hepatic irAEs in real-world settings with over half of patients presenting with ≥ grade 1 AST elevation and 23-27% with ≥ grade 3 liver injury (26, 27).

The incidence of hepatic irAEs appears to increase in patients treated for primary liver cancers, most likely because of the presence of underlying liver disease. While the incidence of ≥ grade 3 ALT elevation in patients treated with Nivolumab for HCC was 8% in an initial trial (Checkmate 040) (28), the reported incidence of a similar increase in major trials for lung cancer was 0% (29–31) and ranged from 0-4% in trials for melanoma (32–35). In a meta-analysis of 117 trials, the incidence of elevated liver enzymes was increased twofold in patients with liver cancer when compared to other solid tumors overall (36), higher than in patients treated for melanoma or non-small cell lung cancer (6). However, this did not lead to an interruption of therapy for HCC patients (6). The initial trials for ICI in HCC after initial systemic therapy (CHECKMATE 040 with nivolumab and KEYNOTE-224 with pembrolizumab), reported ALT elevations of any grade in up to 15% and grade ≥3 in 4-6% of patients (28, 37). However, while 13% of patients required treatment for ILICI, only 3.4% of patients discontinued study participation due to hepatitis (38). Recent trials have evaluated a priming dose of tremelimumab (anti-CTLA-4) with subsequent administration of durvalumab (anti-PDL1) to minimize toxicity: this strategy seems to be associated with favorable results since less than 4% of patients required therapy for hepatic irAEs (39). The combination of ICI with other molecules (such as VEGF inhibitors) has been associated with favorable clinical outcomes, with ALT elevations reported in 14% of cases overall and ≥ grade 3 in 3.6% of cases (40).

In a meta-analysis of 17 clinical trials, CTLA-4 inhibitors had a higher propensity to cause hepatotoxicity than PD-1 inhibitors (Odds ratio for high-grade hepatoxicity 1.52 vs 0.48) (41). In a meta-analysis of 117 trials, any-grade ALT and AST elevation was noted in less than 6% of cases and was slightly higher in anti-PD-1 when compared to anti-PD-L1 therapy with ≥ grade 3 increase in 1.3% of cases (36). In combination therapy, up to a third of patients developed elevated liver enzymes, with ≥ grade 3 hepatitis occurring in 6.4-31% of cases, of which 80% required treatment, representing the most common ≥ grade 3 irAE (2, 3, 42). Forty percent of patients developing irAEs on combination therapy developed another immune event (hepatitis in 36%) or recurrence (hepatitis in 17%) on anti-PD-1 monotherapy (4). In another cohort, irAE recurrence ensued in 34% of patients, with 21% ≥ grade 3 in severity (43). In contrast, other cohorts presented much lower rates with no recurrence of ILICI with retreatment (44).

### irAEs and Pre-Existing Autoimmunity

Data on whether underlying autoimmunity is associated with increased irAEs often stems from retrospective trials. Furthermore, whether ICI induces autoimmunity remains unclear. In a systematic review, only 18% of patients presenting hepatic toxicity had detectable anti-nuclear antibodies, although rates were higher in other organ toxicities (45).

Patients with autoimmune liver disease have not been included in studies of patients with underlying autoimmunity undergoing ICI therapy. Incidence of all-grade irAEs in patients with autoimmune disease ranges from 29 to 45% (46–49). Notably, the occurrence of irAEs did not impact overall survival in these studies (46). Worsening of an underlying autoimmune disorder, or “flares” with increased dermatological, rheumatological, or gastrointestinal symptoms, were reported in 29% to 47% of patients (43, 47, 49). Some authors report that most of these complications were easily managed and discontinuation of therapy was not required. Moreover, clinical response was achieved in 40% of patients (43). Nonetheless, in a cohort of patients with lung cancer or melanoma, active autoimmune disease at baseline and underlying immunosuppression have been associated with shorter median progression-free survival (49).

While the incidence of hepatic irAEs in patients with underlying autoimmune hepatitis has not been reported, the histological findings outlined previously can help in differentiating both conditions. Additionally, ILICI was compared to classical AIH in one study based on clinical characteristics; overall, ILICI patients were older, had normal IgG levels in 94% of cases, and were ANA positive in 25% of cases, as opposed to 84% in AIH. Cases of ILICI often required higher doses of steroids but shorter tapers without the need for additional therapy (44).

### Treatment and Outcomes

Clinical practice guidelines recommend corticosteroid therapy (methyl(prednisolone) 1 mg/kg/day) for grade 2 toxicity with the resumption of checkpoint inhibitor therapy once improvement is noted and corticosteroids tapered. In cases of ≥ grade 3 toxicity, treatment with corticosteroids at higher doses (1-2 mg/kg/day (methyl)prednisolone) is recommended followed by mycophenolate mofetil if no response is achieved (12). To limit the toxicity of steroids, reducing the dose to 1 mg/kg of methylprednisolone for ≥ grade 3 toxicity was reported to produce similar rates of response with fewer side effects (50). In addition, the use of budesonide has been described in case reports to facilitate the re-introduction of immunotherapy (PD-1 antagonists) in patients with grade 3 toxicity (51). Initially, permanent discontinuation of ICI and immunosuppressive treatment was deemed necessary in all cases of ≥ grade 3 irAEs (12). However, management of severe irAEs has evolved over the years and individualized management is recommended based on reports of grade 3 irAEs not requiring corticosteroid therapy with ensuing spontaneous resolution (52). This has led to current practice guidelines recommending ICI re-introduction in patients with asymptomatic grade 3 toxicity and to permanently discontinue only if symptomatic (14). However, in a cohort of patients with severe ILICI, including grade 4 toxicity, re-introduction of ICI therapy (with the same agent in 78% of cases) led to recurrence in 35% of cases (53). In another report, tailored therapy based on the severity of histological and serological hepatitis was successful and spontaneous improvement was noted in 37.5% of patients (20). Recommendations on third-line therapy stem from case reports with T cell-directed therapies using tacrolimus (54) and anti-thymocyte globulins (ATG) (55–57). The use of anti-TNF inhibitors is generally not recommended due to sparse reports of adverse events; however, evidence is limited (58).

ILICI most often resolves in 4 to 6 weeks (13, 52). Protracted biochemical resolution after liver injury has also been described in a case report with a return to near-normal transaminase levels only 5 months after treatment withdrawal (59). Case reports of deaths due to liver failure after ILICI are rare (60), and overall mortality due to ILICI is estimated to be inferior to 0.05% in a systematic review and meta-analysis (61). Nonetheless, ILICI represented 8 to 11% of deaths attributed to ICI-related toxicity in the same cohort, a proportion that remained inferior to deaths due to colitis, pneumonitis, and cardiac toxicity (61). Other repository-based studies have reported higher mortality with hepatotoxicity, although without clear evidence of ILICI in all cases (11).

Overall, a complex equilibrium between tumor response, irAEs, and immunosuppressive therapy has emerged. Currently published reports focus mostly on non-hepatic irAEs and data pertaining specifically to ILICI is lacking. In an initial report, patients developing irAE (all hypophysitis) had improved overall survival; nonetheless, treatment with high-dose steroids led to a reduced survival benefit when compared to low-dose steroids (≤ 7.5 mg prednisone daily) (62). Subsequent reports have highlighted improved anti-tumor efficacy and overall survival in patients with irAEs (63). Meanwhile, baseline corticosteroid use in non-small cell lung cancer for the treatment of neoplasia-related symptoms (notably brain metastases, fatigue, and dyspnea) has been associated with reduced overall and progression-free survival (64). Strikingly, these reports highlight adverse events at steroid doses (7.5 and 10 mg daily) inferior to the recommended doses for treatment of irAEs (62, 64). Therefore, the judicious use of corticosteroids in cases of irAEs might benefit the overall prognosis. Furthermore, a better understanding of the pathophysiology of ILICI might eventually lead to the selection of a more appropriate treatment regimen.

## Etiology of Immune Checkpoint Inhibitors Hepatitis

Several mechanisms have been proposed to explain the presence of ILICI including direct cytotoxicity of the administered antibodies through complement activation (7, 65). While this could be possible since the targets of several cancer immunotherapies, such as PD-1 and PD-L1, are expressed by healthy tissue, it does not explain why the liver would be specifically targeted. Other mechanisms that have also been put forward include epitope spreading in which a diversification of the immune response from the original anti-tumoral response can lead to an indiscriminate autoimmune response (66), a loss of Treg-mediated peripheral tolerance (67) and TNF-α-mediated hepatotoxicity (68). We know that the presence of pre-existing liver disease can increase the incidence of ILICI and that the incidence of hepatic irAEs is increased in patients who received combination therapy (5). However, the specific immune mechanism(s) responsible for the development of ILICI during ICI therapy remain(s) unknown.

The liver’s unique characteristics could be key in understanding the pathogenesis of ILICI. The liver is a very distinct anatomical and immunological site in which blood is constrained through a network of sinusoids with circulating cells being scanned by specialized liver-resident antigen-presenting cells such as Kupffer cells and by highly differentiated liver sinusoidal endothelial cells (LSECs).

### The Liver as an Immunological Organ

The liver has unique immunological features that distinguish it from other non-lymphoid organs (69, 70). Located at the interface between the intestinal and systemic circulations, the liver has evolved specific immune mechanisms to protect the body against pathogens while maintaining a tolerogenic state towards harmless antigens from food and intestinal flora. The distinct microenvironment in which liver-resident immune cells have to function has led to the development of immunological mechanisms unique to the liver.

The liver is the only non-lymphoid organ able to induce the primary activation of naïve CD8+ T cells (71). However, while this activation can lead to fully functional and effective CD8+ T cells (72), it can also lead to tolerance through ineffective activation of CD8+ T cells with defective cytotoxic capacities and shortened half-life (73). This phenomenon is believed to be involved in the development of oral tolerance and the induction of tolerance in liver allografts (70).

The constant exposure of the liver to bacterial lipopolysaccharides (LPS) from the intestinal flora has several consequences on the development of its immune responses. One of these is a phenomenon called “endotoxin tolerance” where exposure of cells to low concentrations of LPS, the natural ligands of TLR4, make them refractory to subsequent stimulation by TLR4 (74). This exposure also leads to the release of immunosuppressive cytokines such as IL-10, TGF-β, HGF (*hepatocyte growth factor*), and retinoic acid by stellate-Ito cells (74). Therefore, LPS exposure leads to the establishment of an immunological microenvironment in the liver that influences the development of subsequent immunological responses.

At the end of an immune response, the population of activated T cells contracts, leaving only a small population of memory T cells. This deletion of CD8+ T cells from the periphery is associated with the accumulation of apoptotic CD8+ T cells in the liver and, in some cases, liver damage (75). This is linked with one of the more interesting effects of LPS on the liver: the induction of low-level expression of adhesion molecules such as ICAM-1 and VCAM-1 by LSECs and Kupffer cells in hepatic sinusoids. ICAM-1 and VCAM-1 adhesion molecules are usually expressed at the sites of inflammation to allow entry into the parenchyma of activated CD8+ T cells. The constitutive expression by the liver of these adhesion molecules is therefore in direct competition with inflamed tissues for the sequestration of circulating activated T cells (76). The ICAM-1 and VCAM-1 expression and the slow blood flow in liver sinusoids that facilitates cellular interactions between circulating activated T cells and LSECs and Kupffer cells, explains the preferential accumulation of activated CD8+ T cells in the liver during the contraction of an immune response (77, 78). This has been shown using a model of experimental activation of CD8+ T cells by either a superantigen, a soluble peptide specific to the T cell receptor, or by an anti-CD3 antibody, which leads to their disappearance from lymphoid organs and the accumulation of apoptotic CD8+ T cells in the liver (79).

While the liver preferentially retains activated CD8+ T cells, CD4+ T cell retention also occurs in the liver but through a different mechanism (76, 80). While activated CD8+ T cell trapping occurs either through VCAM-1/α4β1-integrin or ICAM-1/LFA-1 interaction, activated CD4+ T cells are mostly retained through VCAM-1/α4β1-integrin interactions for Th1 CD4+ T cells, and VAP-1/Siglec-10 for Th2 CD4+ T cells (76, 80). Of note, ICAM-1 and VCAM-1-mediated trapping is responsible for up to 90% of lymphocyte retention in the liver while the remaining 10% is thought to be VAP-1 mediated (76).

This preference for activated CD8+ T cells is due, among other things, to the level of expression of LFA-1, the ICAM-1 receptor (77). LFA-1 is expressed at a much higher level by activated CD8+ T cells than by activated CD4+ T cells (77). In isolated perfused liver experiments, ICAM-1-deficient livers accumulate far fewer activated CD8+ T cells than control livers (77). Additionally, during infection with lymphocytic choriomeningitis virus (LCMV), the migration of activated CD8+ T cells to the liver and other sites of infection is inhibited in the absence of ICAM-1 (81).

Once sequestered by the liver, these activated CD8+ T cells encounter Kupffer cells and, *via* the binding of their FasL molecules to Fas expressed on Kupffer cells and their secretion of IFN-γ, induce the secretion of TNF-α by Kupffer cells (75). TNF-α is then responsible for inducing apoptosis of activated T cells in the liver (75). In a murine model of circulating activated CD8+ T cells, treatment of these mice with anti-TNF-α antibodies led to increased numbers of activated CD8+ T cells in the lymph nodes, spleen, and liver (75). However, apoptosis and caspase activity decreased only in liver CD8+ T cells, but not in lymphoid organs (75). These results indicate that TNF-α is responsible for inducing apoptosis in the liver and suggest that CD8+ T cells escaping this mechanism of deletion can recirculate into the periphery (75).

The secretion of TNF-α during the arrival of massive numbers of activated CD8+ T cells in the liver can cause liver toxicity and lead to hepatocyte death, a phenomenon coined *bystander hepatitis*. This phenomenon can be observed during infection by non-hepatotropic viruses, such as influenza and the Epstein-Barr virus, that induce a strong immune response and the activation of large numbers of T cells (82). This bystander hepatitis, generally moderate or light, can also be observed in other inflammatory diseases such as inflammatory bowel disease or celiac disease given the direct link with the liver through the portal circulation (83, 84). This bystander hepatitis and the associated increase in ALT levels caused by the induction of apoptosis of activated CD8+ T cells is likely directly related to Kupffer cells since their depletion in an experimental model of bystander hepatitis prevents the development of hepatitis (85).

## Proposed Mechanism for Hepatic irAEs in Cancer Immunotherapy

Cancer immunotherapy targets immune checkpoint molecules, namely CTLA-4 and PD-1, that send inhibitory signals that reduce T cell function, activation, and proliferation (86). Therefore, inhibition of these molecules induces an increased activation of T cell immune responses thereby inducing an anti-tumoral response in these patients. It is this increase in circulating activated T cells that have become refractory, at least in parts, to inhibitory signaling due to the ICI blockade therapy that could be responsible for the development of ILICI.

**Figure 1:**
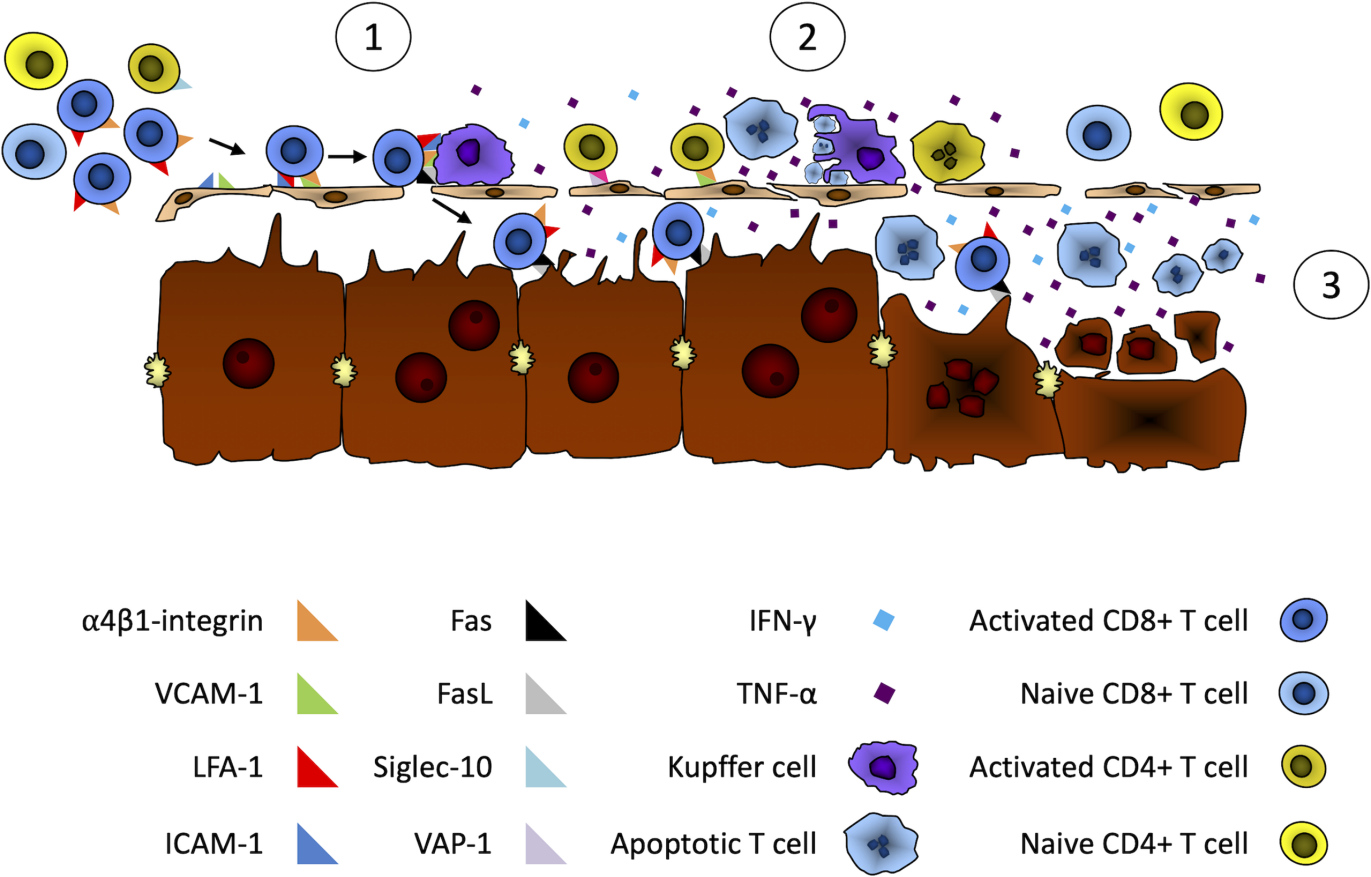
Putative mechanisms of liver damage during immune checkpoint inhibitor cancer immunotherapy. We propose the following series of events leading to liver damage during ICI therapy. 1) Adhesion of activated T cells in hepatic sinusoids. First, activated CD8+ T cells would be trapped by the liver through binding of their α4β1-integrin to LSECs and Kupffer cell-expressed VCAM-1 and ICAM-1. Activated CD4+ T cells could also be retained by the liver through α4β1-integrin and Siglec-10 binding to LSECs- and Kupffer cell-expressed VCAM-1 and VAP-1. 2) Apoptosis of activated T cells. Following their retention in the liver, activated T cells Fas death receptor would bind FasL expressed on LSECs and Kupffer cells. This Fas/FasL interaction and the IFN-gamma secretion by activated CD8+ T cells would induce the expression and secretion of TNF-α by Kupffer cells. This would lead to the apoptosis of activated T cells by both Fas/FasL interactions and the ligation of TNF-α to their *tumor necrosis factor receptor 1* (TNFR1). Apoptosis could also occur through binding of LSECs- and Kupffer cell-expressed TRAIL with *TNF-related apoptosis-inducing ligand receptor* (TRAILR) expressed on activated T cells. 3) Apoptosis of hepatocytes. The activation of Kupffer cells by activated T cells through the combined effect of Fas/FasL ligation and Interferon-gamma secretion by activated T cells would lead to the secretion of large amounts of cytotoxic TNF-α by Kupffer cells. This would sensitize hepatocytes that would then be susceptible to Fas-induced and IFN-gamma-mediated apoptosis by infiltrating activated T cells.

Based on current knowledge of liver immunology, the pathogenesis of AIH, and key observations made about ILICI (65), we propose the following series of events to explain the development of immune-mediated hepatitis following the administration of immune checkpoint inhibitors (**Figure 1**).

### Adhesion of Activated T Cells to Hepatic Sinusoids

Activated T cells generated by ICI therapy would be sequestered by the liver through binding of α4β1-integrin and LFA-1, expressed by activated CD8+ T cells, with adhesion molecules VCAM-1 and ICAM-1, respectively, that are expressed in hepatic sinusoids by LSECs and Kupffer cells (76, 87). Activated CD4+ T cells could also be retained by liver cells through VCAM-1/α4β1-integrin and VAP-1/Siglec-10 interactions for Th1 and Th2 CD4+ T cells respectively (80).

### Apoptosis of Activated T Cells

Following their retention in the liver, activated T cells would interact with Kupffer cells through ligation of Fas death receptor on activated T cells and FasL expressed on LSECs and Kupffer cells. The activation of Fas by FasL and IFN-gamma secretion by activated CD8+ T cells would induce the expression and secretion of TNF-α by Kupffer cells. This would then lead to the apoptosis of activated T cells by Fas/FasL interactions, binding of TNF-α with its cognate receptor *tumor necrosis factor receptor 1* (TNFR1) and LSECs- and Kupffer cell-expressed TRAIL binding with *TNF-related apoptosis-inducing ligand receptor* (TRAILR) expressed by activated T cells (88).

### Apoptosis of Hepatocytes

The activation of Kupffer cells by activated T cells, through the combined effect of Fas/FasL ligation and Interferon-gamma secretion by activated T cells, would lead to increased secretion of cytotoxic TNF-α by Kupffer cells, TNF-α acting as an autocrine amplifier of Kupffer cell function (75). This would sensitize hepatocytes that would then be susceptible to Fas-induced and IFN-gamma-mediated apoptosis by infiltrating activated T cells (89, 90). Hepatocyte injury would also be likely further enhanced due to the immune checkpoint blockade therapy that limits the physiological mechanisms responsible for restricting the immune response against self (PD-1/PD-L1 for example) or inhibiting T cell responses (CTLA-4/B7 for example).

While this is a theoretical model of how an immune-mediated liver injury could occur following ICI treatment, it remains compatible with several observations made during ICI therapy and ILICI. For example, we know that anti-PD-1/anti-CTLA-4 combination treatment induces a profound increase in T cell proliferation and activation (91). Based on the proposed model of ICI hepatitis, this increased number of activated T cells could explain the observed increased incidence of hepatic irAEs in patients with combined anti-PD-1/anti-CTLA-4 treatment (12).

In addition, adhesion molecules such as ICAM-1, VCAM-1, and VAP-1 are known to be over-expressed in the setting of liver inflammation (80, 92, 93). Based on our model, this could impact the sequestration of activated T cells in the liver potentially leading to the increased incidence of ILICI observed in patients with pre-existing liver disease (5, 6).

Interestingly, several observations have linked CD8+ T cells and the development of ILICI including an increased level of perforin- and granzyme B-positive cytotoxic CD8+ T cells in patients with ILICI compared to checkpoint inhibitor-treated patients without ILICI and healthy controls (94, 95), a predominance of CD8+ T cells among liver-infiltrating lymphocytes in patients treated with anti-CTLA4 who developed ILICI (19) and in a murine model of amodiaquine-mediated idiosyncratic drug‐induced liver injury using PD1-/- mice, the depletion of CD8+ T cells prevented liver injury (96).

A key question is why only certain patients treated with ICI will develop ILICI while most do not. Using experimental models, it has been shown that invalidation of immune checkpoint inhibitors, such as PD-1 in PD-1 -/- mice for example, *per se*, does not lead to liver injury (97). However, several drugs, including amodiaquine (96), nevirapine, and isoniazid combined with CTLA4 inhibitors (98) and epigallocatechin gallate (the main catechin in green tea) combined with CTLA4 blockade (99), lead to liver injury. Despite being made in models of idiosyncratic drug‐induced liver injury, these observations suggest that liver injury results from immune checkpoint inhibition combined with a triggering event such as exposure to a drug or xenobiotic. These triggering events could allow the number of activated CD8+ T cells or their level of activation to overcome a threshold necessary to elicit a liver injury. For example, based on our proposed model, a certain number of liver-infiltrating activated CD8+ T cells could be required before a hepatotoxic level of Kupffer cell-secreted TNF-α is reached. This could explain why only certain patients will develop ILICI and others will not.

In this model, we have addressed the most frequent cases of ILICI where liver inflammation is rapidly resolved upon ICI treatment cessation or immunosuppressive treatment. However, there are more severe cases, and given the heterogeneity of liver histology and response to treatment (22, 100), we can postulate that an autoreactive immune response with subsequent autoimmune hepatitis could develop in such conditions. We know that there likely is a triggering event in the development of an AIH in susceptible individuals (101). In refractory cases of ILICI, these patients could have existing circulating autoreactive T cells that, upon ICI treatment, would become activated, possibly through epitope spreading (66), and if they could escape apoptosis in the liver, could have recognized their cognate autoantigen in the liver and proliferated. In these rare cases, the ILICI could have been a trigger and a first step into the development of a *bona fide* AIH. Further research is needed to characterize the immune responses in these patients and determine if a break of immunological tolerance and the development of an autoimmune response can indeed be caused by ICI therapy.

## Conclusion

The success of immune checkpoint inhibitors therapy against several types of cancer has led to their use in an increasing number of patients. Since these treatments are associated with immune-related adverse events, including immune-mediated autoimmune-like hepatitis, these have become an increasing concern for clinicians. The fact that the liver has become one of the targets of these immune-related adverse events is likely in part due to its unique immunological characteristics and microenvironment. Herein, we proposed a series of events, based on the current knowledge of liver immunology and of the pathogenesis of autoimmune hepatitis, that could trigger the liver injury observed in ICI-treated patients. This model could be useful to design further studies aimed at identifying the specific immune mechanism(s) at play in ILICI.

## Author Contributions

JH and PL wrote the first draft of the manuscript. All authors contributed to manuscript revision, read, and approved the submitted version.

## Funding

This review was funded by an Autoimmune Hepatitis Pinnacle Research Award in Liver Disease from the American Association for the Study of Liver Diseases (AASLD) to PL and from a Université de Montréal Novartis/Canadian Liver Foundation Hepatology Research Chair to MB. The funders were not involved in the study design, collection, analysis, interpretation of data, the writing of this article or the decision to submit it for publication.

## Conflict of Interest

The authors declare that the research was conducted in the absence of any commercial or financial relationships that could be construed as a potential conflict of interest.

## Publisher’s Note

All claims expressed in this article are solely those of the authors and do not necessarily represent those of their affiliated organizations, or those of the publisher, the editors and the reviewers. Any product that may be evaluated in this article, or claim that may be made by its manufacturer, is not guaranteed or endorsed by the publisher.
